# Technology advances in diabetes pregnancy: right technology, right person, right time

**DOI:** 10.1007/s00125-024-06216-2

**Published:** 2024-07-05

**Authors:** Anna McLean, Louise Maple-Brown, Helen R. Murphy

**Affiliations:** 1 Menzies School of Health Research, Charles Darwin University, Darwin, NT Australia; 2 Endocrinology Department, Cairns Hospital, Cairns, Queensland Australia; 3 Endocrinology Department, Royal Darwin Hospital, Darwin, Northern Territory Australia; 4 Norwich Medical School, University of East Anglia, Norwich, UK; 5 Norfolk and Norwich NHS Foundation Trust, Diabetes and Antenatal Care, Norwich, UK

**Keywords:** Automated insulin delivery, Closed-Loop, Continuous glucose monitoring, Diabetes technology, Neonatal, Obstetric, Pregnancy, Review, Type 1 Diabetes, Type 2 Diabetes

## Abstract

This review outlines some of the extraordinary recent advances in diabetes technology, which are transforming the management of type 1 diabetes before, during and after pregnancy. It highlights recent improvements associated with use of continuous glucose monitoring (CGM) but acknowledges that neither CGM nor insulin pump therapy are adequate for achieving the pregnancy glucose targets. Furthermore, even hybrid closed-loop (HCL) systems that are clinically effective outside of pregnancy may not confer additional benefits throughout pregnancy. To date, there is only one HCL system, the CamAPS FX, with a strong evidence base for use during pregnancy, suggesting that the pregnancy benefits are HCL system specific. This is in stark contrast to HCL system use outside of pregnancy, where benefits are HCL category specific. The CamAPS FX HCL system has a rapidly adaptive algorithm and lower glucose targets with benefits across all maternal glucose categories, meaning that it is applicable for all women with type 1 diabetes, before and during pregnancy. For women of reproductive years living with type 2 diabetes, the relative merits of using non-insulin pharmacotherapies vs diabetes technology (dipeptidyl peptidase-4 inhibitors, glucagon-like peptide-1 receptor agonists and sodium−glucose cotransporter 2 inhibitors) are unknown. Despite the urgent unmet need and potential benefits, studies of pharmacotherapy and technology use are extremely limited in pregnant women with type 2 diabetes.

**Graphical Abstract:**

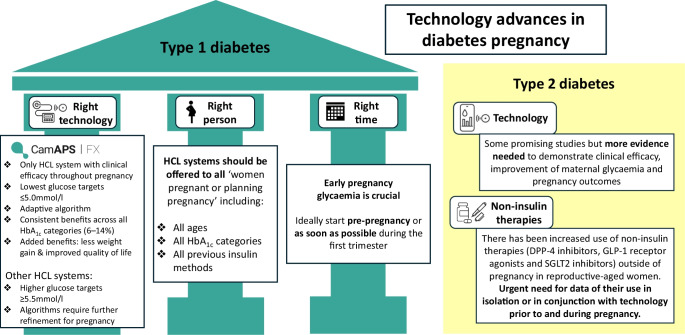

**Supplementary Information:**

The online version contains a slideset of the figures for download available at 10.1007/s00125-024-06216-2.

## Introduction

Thanks to advances in glucose monitoring and insulin therapy, almost 99% of pregnant women with ongoing pregnancies complicated by pre-gestational diabetes now leave hospital with a liveborn baby. This review will outline some of the extraordinary recent advances in diabetes technology, which are transforming the management of diabetes before, during and after pregnancy. We will focus on the right technology for the right person at the right time and examine how some of the current barriers and health inequalities might be overcome. It is worth noting for a global readership that we are using sexed language including the words ‘women’ and ‘mothers’ to ensure that sex-based reproductive health needs are recognised [1]. We also respectfully use the term First Nations to describe Indigenous peoples in a global context and Aboriginal and/or Torres Strait Islander peoples when referring to Australian First Nations people [2].

**Fig. 1:**
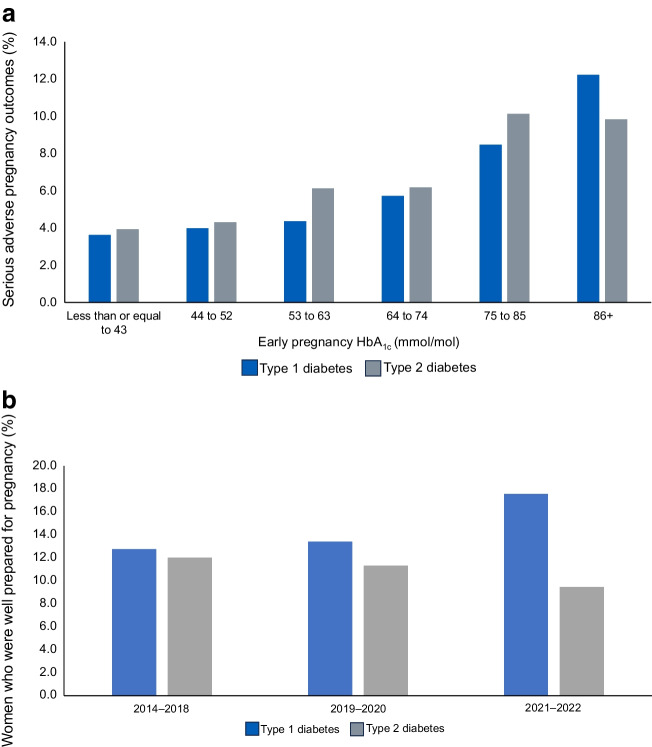
(**a**) Serious adverse pregnancy outcomes (major congenital anomaly, stillbirth, neonatal death) according to early pregnancy HbA_1c_ categories, reproduced from the NPID Audit Report 2020 [5]. (**b**) Widening gaps in pregnancy preparation in type 1 and type 2 diabetes pregnancies, reproduced from the NPID Audit report 2021 and 2022 [14]. This figure is available as part of a downloadable slideset

First, despite numerous scholarly articles about ‘adverse pregnancy outcomes’, for most women with pre-existing (and gestational) diabetes, pregnancy outcomes have never been better. Women and clinicians should be reassured that 95% of women with diabetes have successful pregnancy outcomes, meaning that after excluding early pregnancy losses and miscarriages (for which data are limited), 95% deliver liveborn babies without major congenital anomalies. Population-based data from the UK National Pregnancy in Diabetes (NPID) audit demonstrate that 98.8% of all registered births in mothers with diabetes were livebirths, compared with 99.6% in general maternity population [3]. Whilst the prevalence of serious adverse pregnancy outcomes (major congenital anomaly, stillbirth and neonatal death) remains two to three times higher compared with the general maternity population and can affect up to one in ten unplanned pregnancies with higher HbA_1c_, contemporary UK data are largely reassuring (Fig. 1a). Overall rates of major congenital anomaly are approximately 45 per 1000 births, stillbirth 10–13 per 1000 births and neonatal death 7–11 per 1000 births. Data from 2021 to 2022 report further improvements in type 1 diabetes pregnancies, and suboptimal glycaemia associated with concerning increases in perinatal deaths during type 2 diabetes pregnancy [3].

It is long established that the risk of serious adverse pregnancy outcomes can be minimised by improving pregnancy preparation [4]. This means supporting women to use safe effective contraception until they reach the pregnancy glucose target of HbA_1c_ <48 mmol/mol (6.5%) or as near as possible, taking preconception folic acid and avoiding potentially harmful medications. However, reaching target HbA_1c_ remains extremely challenging among women from younger age groups, higher BMI categories and deprived groups [3]. Pre-pregnancy care services, which depend on women proactively planning pregnancy with specialist teams, are particularly inadequate in women from deprived and ethnic minority groups, most notably in Black women [5]. The socioeconomic gradient is striking, with approximately twice as many well-prepared pregnancies in the least deprived group compared with the most deprived group, and this is observed in both women with type 1 diabetes (41% vs 17%) and women with type 2 diabetes (35% vs 17%). Recent UK data suggests improvements in periconception glycaemia among women with type 1 diabetes, most likely attributed to increasing diabetes technology use during 2021–22, without improvement in type 2 diabetes (Fig. 1b). Furthermore, 12% of women with type 2 diabetes conceived whilst taking treatments for blood pressure or lipids, or newer therapies that are not approved for use during pregnancy, which, alongside inadequate attention to glycaemia and folic acid supplementation, contribute to widening healthcare inequalities [5].

**Fig. 2:**
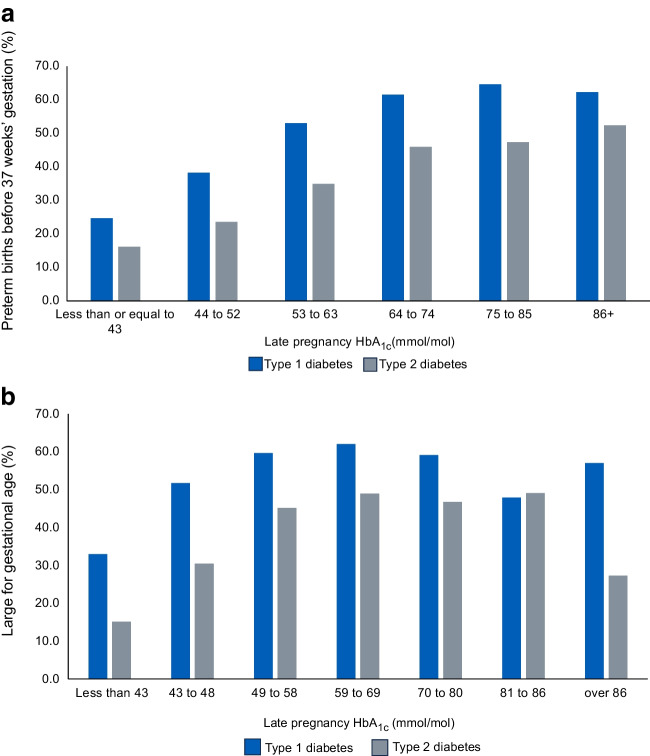
(**a**) Preterm birth (before 37 weeks’ gestation) rates in type 1 and type 2 diabetes pregnancies according to maternal HbA_1c_ categories. (**b**) LGA rates in type 1 and type 2 diabetes pregnancies according to maternal HbA_1c_ categories. Late pregnancy HbA_1c_ is defined as HbA_1c_ from 24 weeks’ gestation (reflecting antenatal glycaemia from approximately 16 to 20 weeks onwards). Reproduced from the NPID Audit report 2020 [5]. This figure is available as part of a downloadable slideset

However, whilst 95% of mothers have successful liveborn babies, obstetric and neonatal complications related to maternal hyperglycaemia remain ubiquitous, affecting one in two and one in three pregnancies complicated by type 1 diabetes or type 2 diabetes, respectively (Fig. 2a, b) [3]. These include preterm births (delivery before 37 weeks’ gestation), large for gestational age (LGA) birthweight (>90^th^ percentile) and neonatal care unit admissions, which separate mothers and babies, thereby interrupting bonding and infant feeding [5]. Whilst most neonatal care unit admissions involve management of easily treated conditions, e.g. transient respiratory distress, neonatal hypoglycaemia or jaundice, these are nonetheless stressful for women and families and costly for healthcare providers. The inter-generational, longer-term impacts on the metabolic health of children exposed to in-utero hyperglycaemia are also important [2, 6, 7]. The longer-term impacts on offspring extend beyond the established conditions of obesity, diabetes and cardiovascular disease, with a growing body of evidence suggesting increased vulnerability to anxiety, depression and autism spectrum disorders [8, 9].

## Continuous glucose monitoring

The CONCEPTT trial provided strong evidence that using continuous glucose monitoring (CGM) during type 1 diabetes pregnancy improves maternal glucose levels and reduces obstetric and neonatal complications [10]. CGM use was clinically effective and cost-saving for healthcare providers because of the reductions in the frequency and duration of neonatal care unit admissions. Based on these data, the UK National Institute for Health and Care Excellence (NICE) recommend that all pregnant women with type 1 diabetes are offered CGM [11]. CGM use has increased in many countries, such as the increase from 25% to 45% among recent participants in the USA type 1 diabetes exchange registry clinics; however, age, racial and socioeconomic barriers were prevalent [12]. In the UK, national implementation of CGM use was accelerated by ring-fenced funding provided to local maternity services, which transformed the clinical management of type 1 diabetes pregnancy [13]. By 2022, 95% of pregnant women with type 1 diabetes were using CGM (75% real-time CGM, 20% intermittently scanned CGM), with fewer than 5% using fingerstick blood glucose monitoring [5, 13, 14].

**Fig. 3:**
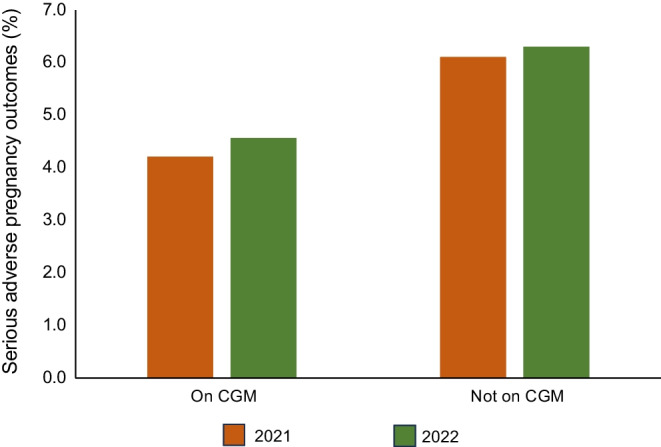
Serious adverse pregnancy outcomes (major congenital anomaly, stillbirth, neonatal death) according to CGM use during type 1 diabetes pregnancies in 2021–2022. CGM users had reduced odds for serious adverse pregnancy outcomes (OR 0.70 95% CI 0.53, 0.94; *p*=0.015). Reproduced from the NPID Audit report 2021 and 2022 [14]. This figure is available as part of a downloadable slideset

Real-world data are now available for 2055 type 1 diabetes pregnancies (825 in 2021, 1230 in 2022) where CGM was used before and/or during pregnancy (Table 1). CGM users had small but significant improvements in periconception glucose levels, with slightly more achieving target HbA_1c_ in early pregnancy [14]. However, glycaemic benefits were more apparent during pregnancy, with significantly more CGM users achieving target HbA_1c_ after 24 weeks’ gestation. Glycaemic improvements were accompanied by fewer maternal hospital admissions for diabetic ketoacidosis (DKA), and without additional severe hypoglycaemia. Obstetric and neonatal benefits included fewer preterm births, LGA babies and neonatal care admissions [14]. Whilst differences in neonatal complications are modest, important additional benefits included reduced odds of serious adverse pregnancy outcomes in CGM users (OR 0.70 95% CI 0.53, 0.94; *p*=0.015) (Fig. 3). Furthermore, rates of perinatal deaths were also significantly lower, reflecting improved antenatal glycaemia during pregnancy, whilst rates of congenital anomaly were numerically (2.9% vs 3.8%) but not statistically significantly lower, perhaps suggesting smaller benefits in pre-pregnancy glycaemia. Thus, after many years without progress, and rising rates of neonatal complications, CGM use was associated with real-world improvements in type 1 diabetes pregnancy outcomes, across an entire population [5]. However, despite national funding there were some unexpected socioeconomic and ethnic disparities. Women living in the most deprived areas and those using multiple daily injections were more likely to be given intermittently scanned vs real-time CGM, suggesting systemic barriers that should be addressed for future implementation of diabetes technologies.

**Table 1:** Real-world data for type 1 diabetes pregnancies in the UK where CGM was used before and/or during pregnancy

Pregnancy outcomes^a^	CGM users	Non-CGM users
Target HbA_1c_ <48 mmol/mol (6.5%) during early pregnancy	25.5%	22.4%
Target HbA_1c_ <43 mmol/mol (6.1%) after 24 weeks’ gestation	35.1%	25.3%
Maternal hospital admission for diabetic ketoacidosis (DKA) events	2.2%	2.9%
Preterm births <37 weeks’ gestation	39.5%	43.9%
LGA babies	45.6%	53.5%
Neonatal care unit admissions	44.8%	48.5%
Major congenital anomaly	2.9%	3.8%
Perinatal deaths	1.7%	2.6%
Serious adverse pregnancy outcomes	4.4%	6.2%

This table has been prepared using data from the UK NPID audit [14], which included *N*=2055 pregnancies (825 in 2021, 1230 in 2022)

^a^Apart from major congenital anomaly, which did not reach statistical significance, all other between-group differences for CGM vs non-CGM users are statistically significant (*p*<0.05). Most notable is that CGM users had reduced odds for the composite serious adverse pregnancy outcomes (major anomaly and/or perinatal death) (OR 0.70 95% CI 0.53, 0.94; *p*=0.015)

The NPID audit reports do not include *p* values, but data are publicly available at https://digital.nhs.uk/data-and-information/publications/statistical/national-pregnancy-in-diabetes-audit

## What next?

Further improvements in maternal glucose levels and reductions in complications attributed to maternal hyperglycaemia will require more physiological approaches, including glucose responsive insulin replacement therapy in type 1 diabetes pregnancy [15]. Achieving the stringent pregnancy glucose targets of HbA_1c_ <43 mmol/mol (6.1%) and ≥70% of sensor glucose values in the pregnancy-specific target range (i.e. time in range for pregnancy [TIRp] 3.5–7.8 mmol/l or 63–140 mg/dl) is accomplished by only ~33% of CGM users, regardless of insulin delivery method (pump or multiple daily injections) [16]. Furthermore, even the newer generation insulin analogues, which are clinically effective outside of pregnancy and safe for use during pregnancy, are insufficient for optimal antenatal glycaemia [17, 18]. Pregnancy is a dynamic state of continuous metabolic adaptation, with striking changes in insulin sensitivity and insulin pharmacokinetics both on a day-to-day and weekly basis. We have shown that systemic glucose disposal is markedly delayed, leading to more prolonged postprandial hyperglycaemia, during the second and third trimesters [19]. Subcutaneous insulin absorption is also increasingly delayed (almost 50% slower at 38 weeks) and significantly more variable during late pregnancy [20]. Because the developing fetus is uniquely susceptible to maternal hyperglycaemia, TIRp targets are particularly stringent, demanding an additional 5–6 h/day (20–25%) of sensor glucose values in the target range, since 70% TIR 3.9–10 mmol/l (70–180 mg/dl) represents approximately only 45–50% TIRp [21, 22]. The strong moral mandate and sense of utmost responsibility to protect their babies is associated with extraordinary vigilance and unprecedented mental burden for pregnant women: ‘every reading you see, you think, “oh my God, I’m harming the baby”’ [23].

## Hybrid closed-loop systems

Outside of pregnancy, hybrid closed-loop (HCL) systems are increasingly used in type 1 diabetes management [24, 25]. They are clinically effective across a range of adult and paediatric populations for safely improving glycaemia and minimising the mental burden of diabetes management [26–28]. Importantly, there is no evidence for differences between different HCL systems, suggesting that benefits are HCL *category specific* outside pregnancy [29].

Hitherto, trials of HCL systems during pregnancy were limited in size and scope, with short duration single-arm studies, small case series using off-label HCL systems, or early-generation devices with technical glitches requiring more extensive user and healthcare professional input [30]. Whilst small scale randomised trials showed promise, they were not sufficiently compelling to change clinical guidelines [31–33].

The Automated insulin Delivery Amongst Pregnant women with Type 1 diabetes (AiDAPT) trial provided landmark evidence supporting use of a uniquely adaptive HCL system (CamAPS FX) across a generalisable patient population [22]. The trial recruited 124 women (age range 19.7 to 44.7 years, BMI 18.0 to 48.9 kg/m^2^ and baseline HbA_1c_ ranging from 42 mmol/mol to 130 mmol/mol [6.0% to 14%]), more than half of whom were pump naive. Sixty-three were randomised to CGM (Dexcom G6) alongside their usual insulin therapy, and 61 to the HCL system. All other aspects of diabetes pregnancy care (education, clinic visits, fetal surveillance) were standardised in accordance with NICE clinical guidance. The biomedical results were striking, with users of the HCL system spending 10.5 percentage points more TIRp from 16 weeks’ gestation until delivery. Further glycaemic benefits included 10% less time hyperglycaemic, 12% more overnight TIRp and less time in hypoglycaemia overnight throughout pregnancy. Users of the HCL system had clinically relevant glycaemic improvements from the first trimester (5% TIRp), apparent within days of starting to use the HCL system, and these improvements were consistently maintained until delivery. Unanticipated maternal health benefits included 3.7 kg (8lbs) less gestational weight gain and a reduction in hypertensive pregnancy disorders. Whilst underpowered to detect between-group differences in pregnancy outcomes, rates of LGA were substantially lower in babies of HCL system users than comparable type 1 diabetes studies or NPID population data [3, 17, 18], suggesting potential for further real-world population benefits. As more HCL systems become available, some licensed for use in pregnancy and others used ‘off-label’, we should consider which HCL systems offer clinically relevant improvements in maternal glucose outcomes, and which is the right technology for the right person at the right time.

### Which HCL technology is most effective for use during pregnancy?

The AiDAPT results are applicable only to the CamAPS FX system, which has an adaptive algorithm (adapting over 24 h, after meals and day-to-day), lower glucose targets (AiDAPT participants used 5.4 mmol/l [97 mg/dl] and 5.1 mmol/l [93 mg/dl] in the second and third trimesters, respectively) and is the most extensively studied system in type 1 diabetes pregnancy [31, 32]. Thus, the benefits of CamAPS FX use cannot be extrapolated to HCL systems with higher glucose targets or less adaptive algorithms, which require additional user inputs, e.g. ‘fake’ carbohydrates to compensate for increasing post-meal insulin requirements [34, 35]. The CRISTAL trial using the Medtronic 780G HCL system showed a striking lack of clinically relevant glycaemic benefits, with no improvement in TIRp, mean glucose or hyperglycaemic metrics [36, 37]. Use of insulin pump therapy and higher third trimester insulin doses are associated with excessive gestational weight gain, so using HCL systems, which further increase insulin doses, contributing to gestational weight gain and higher rates of LGA, has immediate and longer-term health implications for both mother and baby [38, 39]. Real-world data from Spain showing no glycaemic improvements for pregnant women using other (Medtronic 780G, Tandem Control IQ and Diabeloop) HCL systems are concerning, with HCL system users, particularly those with HbA_1c_ >48 mmol/mol (6.5%), gaining more weight and having heavier newborns [39]. Together with the AiDAPT and CRISTAL studies, this suggests that during pregnancy, the benefits of using HCL technology are *system specific* rather than *category specific*.

### Which women benefit most from using HCL systems?

Outside of pregnancy, both randomised trial and real-world data point to maximal benefits in those with higher baseline HbA_1c_. However, AiDAPT HCL system users had benefits across all maternal glucose categories (7.5% higher TIRp with baseline HbA_1c_ 43–53 mmol/mol [6–7%], 10.9% higher TIRp with HbA_1c_ 53–64 mmol/mol [7–8%] and 11.9% with HbA_1c_ >64 mmol/mol [>8%]), all clinical sites, and regardless of previous diabetes technology use [22, 40]. The consistent biomedical benefits were supported by qualitative data from women, including those from diverse social backgrounds, for whom being able to continue working was crucially important: ‘Honestly, it allowed me to work. I would never be able...to work at the job that I was doing [waitressing] at all, if I didn’t have the machine’ [23]*.* Thus, the NICE clinical guidelines, based on the AiDAPT results, recommend that HCL systems should be offered to *all* ‘women, trans men and non-binary people with type 1 diabetes who are pregnant or planning to become pregnant’[41].

### When is the best time to start using an HCL system?

Since glycaemic control in early pregnancy is the key predictor for serious adverse pregnancy outcomes, use of an HCL system would ideally be started before pregnancy, thus allowing more women to enter pregnancy with near-target glycaemia. However, given the health inequalities between women who do and do not plan pregnancy, starting to use an HCL system as soon as possible after confirmation of pregnancy is likely to be the most effective means of reducing obstetric and neonatal complications in unplanned pregnancies. Analysis of CGM profiles suggest that optimising maternal glucose by 10 to 12 weeks’ gestation is key to preventing the stagnation and/or deterioration of antenatal glycaemia in mid-gestation that is strongly associated with fetal growth acceleration and LGA birthweight [42, 43]. The current evidence suggests that the CamAPS FX HCL system is the most effective means of rapidly optimising maternal glucose in early pregnancy [22]. Other commercially available HCL systems did not improve glycaemia until the final weeks of pregnancy, which is too late for optimal pregnancy outcomes [36, 37, 39].

Pregnancy teams must now consider how to effectively implement the CamAPS HCL system, and how to educate wider healthcare teams at scale, including among smaller sites without specialist teams as well as non-diabetes specialists in emergency departments and maternity units. Qualitative research findings suggest that optimal clinical benefits require engaged users, system-specific training and healthcare teams with sufficient technical know-how to support collaborative working between women, the technology and wider healthcare teams [44]. Furthermore, we should examine larger real-world datasets to examine pregnancy outcomes and ensure equitable access to this life-changing technology.

## Rise in early-onset type 2 diabetes

The global epidemic of type 2 diabetes in younger people has contributed to an increase in type 2 diabetes during pregnancy, particularly among First Nations women and other marginalised population groups [45, 46]. Primary care professionals and obstetric physicians are now more likely to see type 2 diabetes than type 1 diabetes in pregnancy, with or without tertiary endocrinologist input, depending on the local healthcare model. Additional challenges of pregnancy care for women with type 2 diabetes relate to their greater socioeconomic deprivation, poorer social determinants of health and increased prevalence of comorbidities such as hypertension, smoking and obesity [3, 47, 48]. Geographic remoteness has also been shown to negatively affect pregnancy outcomes of women with pre-existing diabetes [48, 49].

Recent data confirm a shift in the management of type 2 diabetes, with increasing use of second-line non-insulin therapies (dipeptidyl peptidase-4 [DPP-4] inhibitors, glucagon-like peptide-1 [GLP-1] receptor agonists and sodium−glucose cotransporter 2 [SGLT2] inhibitors) among women of reproductive age [3]. The increased use of GLP-1 receptor agonists is particularly striking, most likely due to their beneficial effects on weight reduction. Whilst pregnancy outcome data remain limited, they are largely reassuring. A large cohort study that included 50,000 type 2 diabetes pregnancies across six countries (USA, Finland, Iceland, Norway, Sweden and Israel) found no strong evidence for increased rates of major congenital anomaly, or of cardiac malformations, associated with use of GLP-1 receptor agonists compared with insulin therapy [50]. This calls into question the conventional clinical practice of transferring women to insulin therapy before conception, suggesting that the benefits of continuing GLP-1 receptor agonist therapy use until confirmation of a positive pregnancy test may outweigh potential concerns regarding teratogenicity. One third of those with newly diagnosed diabetes are women of reproductive age, so more data regarding the role of diabetes technology vs non-insulin pharmacotherapy before and during pregnancy are urgently needed.

Maternal glucose is by far the strongest potentially modifiable risk factor for stillbirth and neonatal death. Women with type 2 diabetes have higher rates of perinatal death compared with those with type 1 diabetes (OR 1.65), with a substantial negative impact of deprivation (OR 2.29) for living in the most vs least deprived regions, but having a third trimester HbA_1c_ >48 mmol/mol (6.5%) is the strongest predictor for perinatal death (OR 3.06) [3].

## Technology in early-onset type 2 diabetes

Despite the urgent need and potential benefit of diabetes technology use for women with type 2 diabetes, studies are scarce. A systematic review and meta-analysis of CGM use in type 2 diabetes pregnancy published in 2023 included only two RCTs, with a total of 56 participants with type 2 diabetes pregnancy [51]. Effectiveness of CGM compared with fingerstick glucose monitoring initially showed promise, with one early trial of masked CGM (in participants with either type 1 diabetes or type 2 diabetes) being associated with lower third trimester HbA_1c_ levels and less macrosomia [52]. Others have shown that CGM appears to be safe and comparable to fingerstick monitoring in type 2 diabetes pregnancy [51, 53–55], but have lacked statistical power to examine effectiveness on pregnancy outcomes [53, 56, 57].

In a pilot study of 57 Aboriginal and Torres Strait Islander and multi-ethnic women in regional and remote Northern Australia, we showed that intermittently scanned CGM (Freestyle Libre 1) was feasible and preferred over fingerstick monitoring by high-risk women with type 2 diabetes pregnancy [55]. The majority of participants found CGM acceptable, worthwhile and easy to use, and 94% would recommend CGM use to others. Improvements in knowledge and self-management supported the use of CGM as an educational tool [58]. Ethnicity and remoteness were not barriers to CGM use when freely available to all women, despite the previously recognised racial/ethnic disparities that exist in CGM uptake for non-pregnant populations with type 1 diabetes [59–61].

Remote monitoring of CGM levels by health professionals was beneficial during the COVID-19 restrictions and these virtual care models have persisted [59]. Potential benefits of CGM use in type 2 diabetes pregnancy include improved maternal wellbeing and increased fingerstick glucose monitoring [55]. However, effectiveness for supporting implementation of a healthy lifestyle, including limitation of weight gain and improving maternal glucose levels and pregnancy outcomes, has not been established. A multicentre RCT of the clinical- and cost-effectiveness of using CGM in 422 pregnant women with type 2 diabetes is ongoing. The PRegnancy Outcomes using continuous glucose monitoring TEChnology in pregnant women with early-onset Type 2 diabetes (PROTECT) trial will examine whether CGM use is effective for improving TIRp and reducing neonatal care admission or perinatal death (ISRCTN12804317).

It is important to note that women’s experiences with complex insulin regimes and intense glucose monitoring prior to pregnancy are likely to be very different for individuals with type 2 diabetes compared with those with type 1 diabetes. Challenges unique to type 2 diabetes pregnancy include the short time window to train health professionals and women; difficulty with access, cost and late referrals; and the potential for overwhelming the woman with excess information, leading to added emotional or behavioural burdens [55, 62]. Discomfort, skin irritation, pharmacologic interference, alarm fatigue, inaccuracy in the low blood glucose range and discontinuation are pitfalls of CGM sensor use for anyone with type 1 diabetes or type 2 diabetes [62]*.*

More consistent CGM sensor use can improve maternal glucose levels, as supported by our recent findings that only those with increased sensor activity time benefitted from improved glycaemia throughout pregnancy [53, 63]. The variable intermittent use of sensors and high discontinuation rate (20%) in our cohort of high-risk women has implications for the possible future use of CGM, insulin pumps and other diabetes technologies, including HCL systems. Not all patients have compatible smart phones, or enough mobile data or internet access, for all CGM sensors to suit all women.

## Sensor glucose targets in type 2 diabetes pregnancy?

It is now accepted that an increase of 5% TIRp during the second and third trimester is associated with reduced risk of LGA and neonatal hypoglycaemia [64]. Although our CGM dataset in type 2 diabetes pregnancy is small, it nonetheless demonstrated that every 1% higher TIRp in early pregnancy was associated with 4% lower risk of LGA birthweight, similar to type 1 diabetes [56, 63, 65], supporting the need for optimising early pregnancy glycaemia in type 2 diabetes pregnancy [42]. Our pilot study in a high-risk cohort demonstrated alarming rates of neonatal complications in the context of persistent maternal hyperglycaemia throughout pregnancy. Neonatal hypoglycaemia was associated with nearly all CGM metrics, HbA_1c_ levels and TIRp target attainment in early and in late pregnancy.

The 2019 International Consensus on Time in Range acknowledged that more data are required to demonstrate how CGM metrics relate to and predict clinical outcomes in type 2 diabetes pregnancy [66]. We think it is unlikely that the 70% TIRp recommendations used for type 1 diabetes will be applicable in type 2 diabetes pregnancy. Since women with type 2 diabetes enter pregnancy with higher TIRp, and have more rapid first trimester optimisation [67], they should possibly have higher TIRp targets (e.g. 85–90% TIRp). Alternatively, a more stringent TIRp target range (3.5–6.7 mmol/l or 63–120 mg/dl) may be applicable for type 2 diabetes and gestational diabetes mellitus (GDM) pregnancies. Consensus targets for other CGM metrics such as average glucose and glucose management indicator may also be applicable. The PROTECT trial will further examine the associations between maternal CGM metrics with type 2 diabetes outcomes so that appropriate TIRp and mean glucose targets can be established. Ongoing research will inform the development of CGM-based targets in healthy pregnancy and in GDM pregnancy. A better understanding of the changes in CGM profiles throughout healthy and GDM pregnancy is also needed to inform GDM management. Data from two large prospective studies (MAGIC and GLAM) will potentially pave the way for earlier diagnosis of GDM, based on CGM glucose metrics from the first trimester.

## Conclusion

These are exciting times with substantial improvements in maternal glucose outcomes, associated with the increasing use of diabetes technologies before and during pregnancy. It is imperative that women with diabetes who are of reproductive age are a priority for health providers, and that culturally appropriate systems of care are in place to best support optimal technology use before, during and after pregnancy. Pregnant women with type 1 diabetes should be informed that CamAPS FX is the only HCL system with robust evidence of clinical benefit, and that continued use of commercially available HCL systems with higher glucose targets or less adaptive algorithms may be associated with stagnant glycaemia, higher maternal and higher neonatal weight gain. Ensuring adequate access to diabetes educators and expertise across regional and remote areas may assist those with type 2 diabetes to consistently use technology [55]. Improving communication, workforce capacity and skills, health literacy of both health professionals and women, and ensuring culturally appropriate education are imperative for optimal diabetes technology use [63]. Working in partnership to raise the voices of marginalised and disadvantaged communities, particularly women with lived experience of type 2 diabetes pregnancy, is critical to address the increasingly inequitable health outcomes [68–70].

## Supplementary Information

Below is the link to the electronic supplementary material.

ESM slideset (PPTX 280 KB)

## Abbreviations

AiDAPT: Automated insulin Delivery Amongst Pregnant women with Type 1 diabetes
CGM: Continuous glucose monitoring
GDM: Gestational diabetes mellitus
GLP-1: Glucagon-like peptide-1
HCL: Hybrid closed-loop
LGA: Large for gestational age
NICE: National Institute for Health and Care Excellence
NPID: National Pregnancy in Diabetes
PROTECT: PRegnancy Outcomes using continuous glucose monitoring TEChnology in pregnant women with early-onset Type 2 diabetes
TIRp: Time in range for pregnancy

## Authors’ relationships and activities

HRM serves on the Medtronic European and the Ypsomed Scientific Advisory Boards, reports research devices at reduced and no cost from Abbott Diabetes Care (no cost), Dexcom (reduced cost) and Medtronic (no cost), speaker honoraria from Abbott Diabetes Care, Dexcom, Eli Lilly, Medtronic, Novo Nordisk, Sanofi and Ypsomed. She chairs the National Pregnancy in Diabetes (NPID) audit and is a member of the editorial boards for Diabetes Care and Diabetologia journals. AM and LM-B declare that there are no relationships or activities that might bias, or be perceived to bias, their work.
