# Supplementary material for: Technology advances in diabetes pregnancy: right technology, right person, right time
Source: Diabetologia. 2024 Jul 5;67(10):2103–13. doi: 10.1007/s00125-024-06216-2 (PMC11447080; doi:10.1007/s00125-024-06216-2)
Supplement: Supplementary file 1 — ESM slideset (PPTX 280 KB) [file 125_2024_6216_MOESM1_ESM.pptx]

## Slide 1
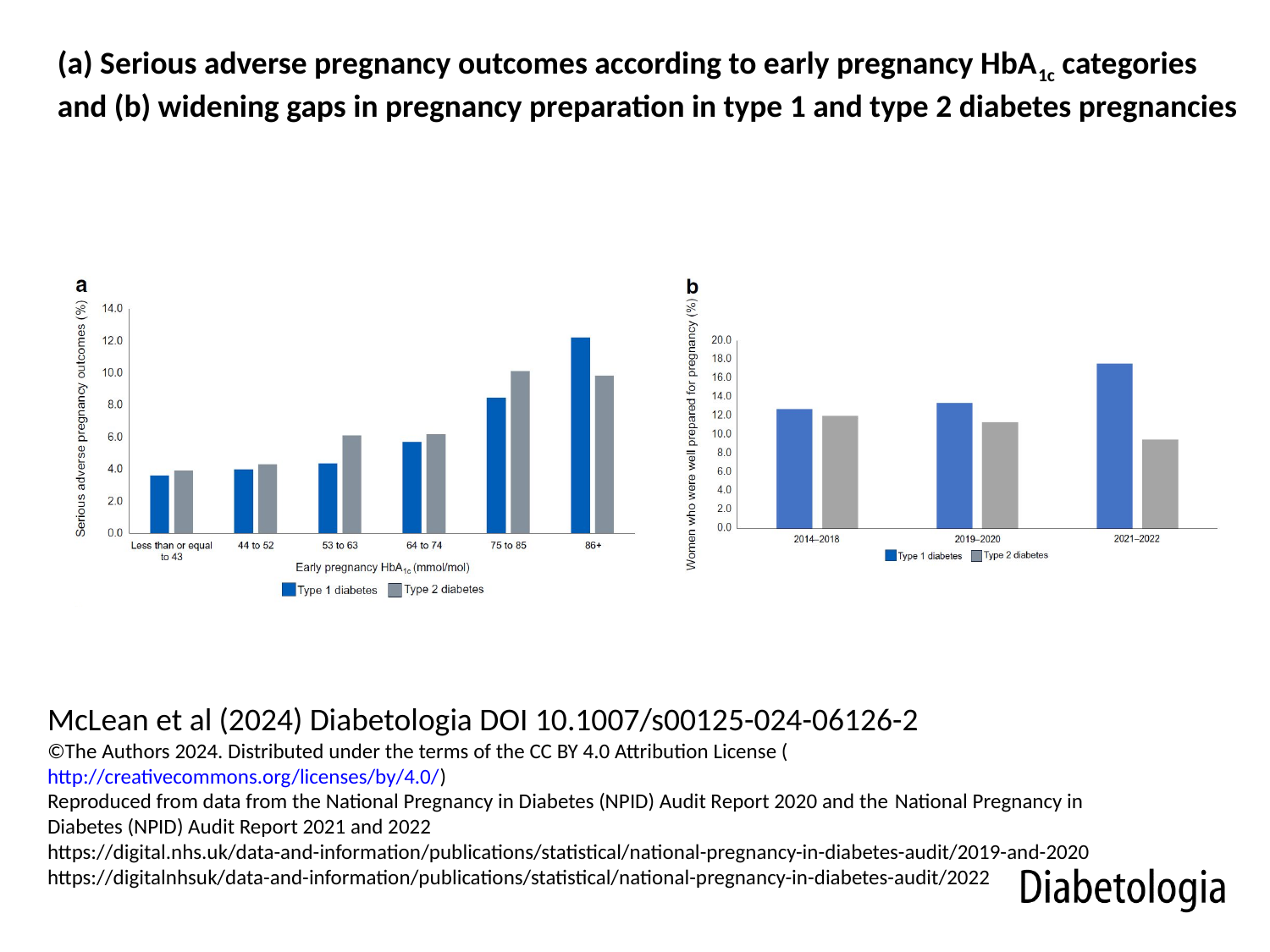

(a) Serious adverse pregnancy outcomes according to early pregnancy HbA1c categories and (b) widening gaps in pregnancy preparation in type 1 and type 2 diabetes pregnancies
McLean et al (2024) Diabetologia DOI 10.1007/s00125-024-06126-2
©The Authors 2024. Distributed under the terms of the CC BY 4.0 Attribution License (http://creativecommons.org/licenses/by/4.0/)
Reproduced from data from the National Pregnancy in Diabetes (NPID) Audit Report 2020 and the National Pregnancy in Diabetes (NPID) Audit Report 2021 and 2022
https://digital.nhs.uk/data-and-information/publications/statistical/national-pregnancy-in-diabetes-audit/2019-and-2020
https://digitalnhsuk/data-and-information/publications/statistical/national-pregnancy-in-diabetes-audit/2022

## Slide 2
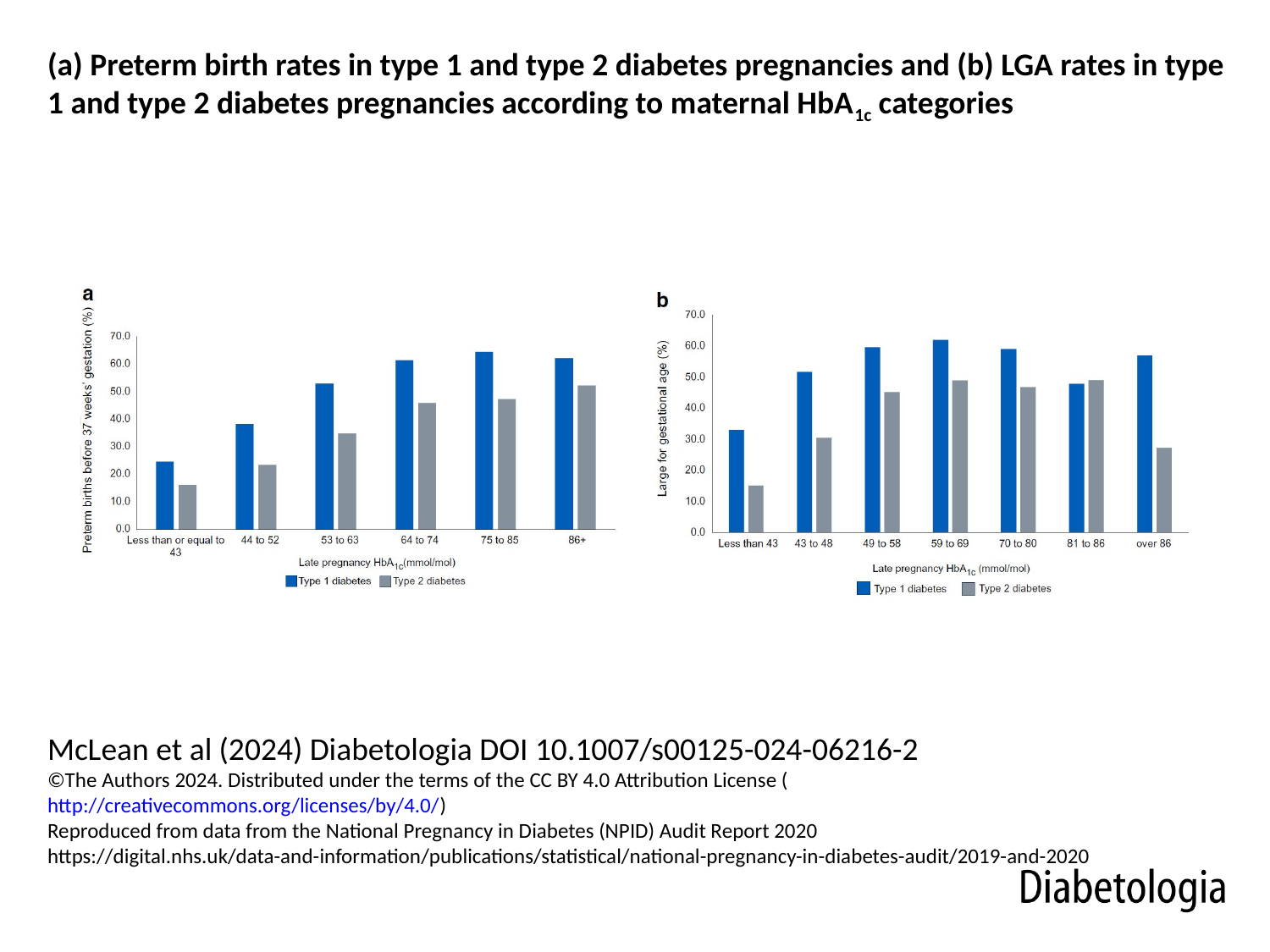

(a) Preterm birth rates in type 1 and type 2 diabetes pregnancies and (b) LGA rates in type 1 and type 2 diabetes pregnancies according to maternal HbA1c categories
McLean et al (2024) Diabetologia DOI 10.1007/s00125-024-06216-2
©The Authors 2024. Distributed under the terms of the CC BY 4.0 Attribution License (http://creativecommons.org/licenses/by/4.0/)
Reproduced from data from the National Pregnancy in Diabetes (NPID) Audit Report 2020
https://digital.nhs.uk/data-and-information/publications/statistical/national-pregnancy-in-diabetes-audit/2019-and-2020

## Slide 3
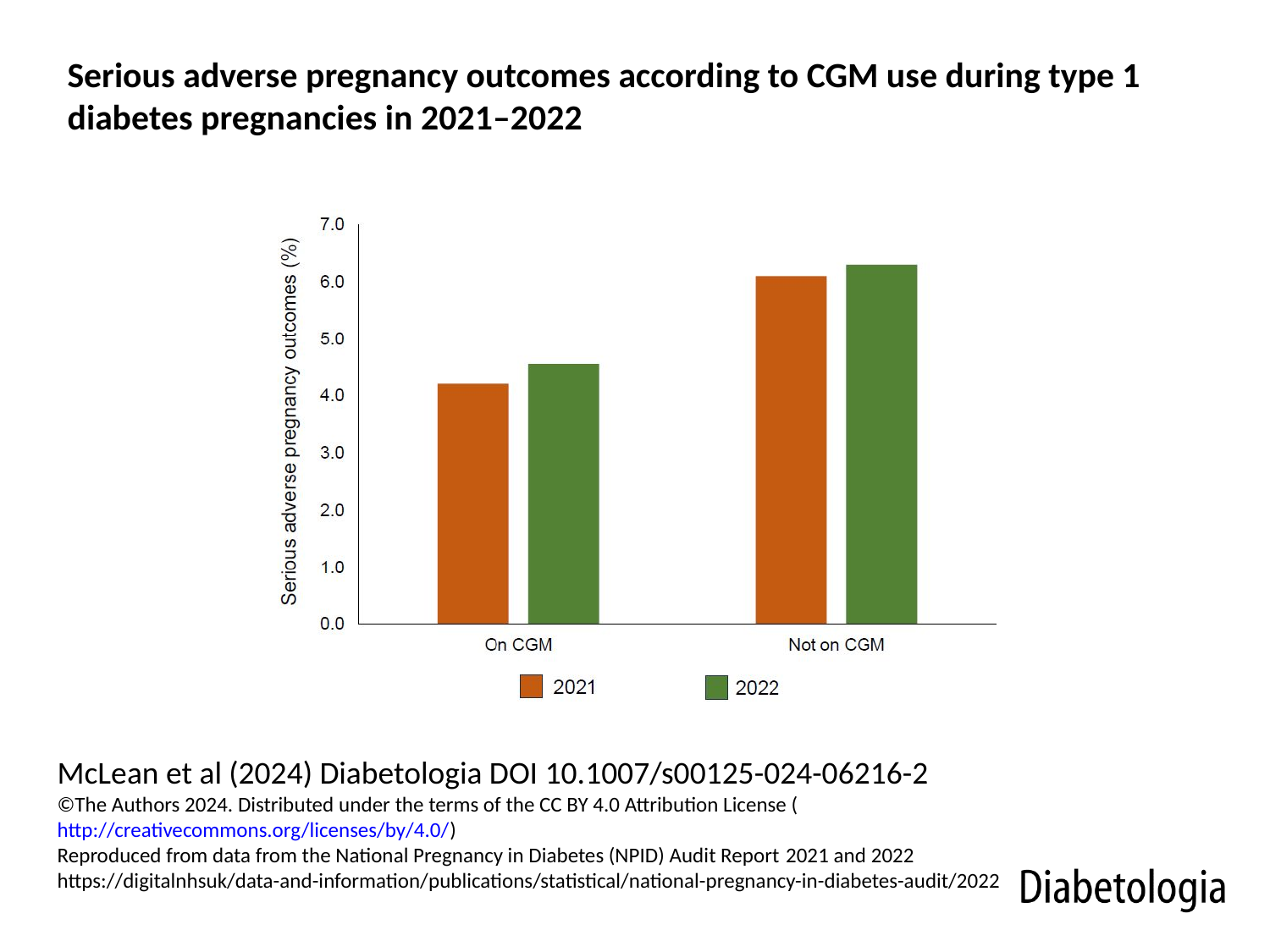

Serious adverse pregnancy outcomes according to CGM use during type 1 diabetes pregnancies in 2021–2022
McLean et al (2024) Diabetologia DOI 10.1007/s00125-024-06216-2
©The Authors 2024. Distributed under the terms of the CC BY 4.0 Attribution License (http://creativecommons.org/licenses/by/4.0/)
Reproduced from data from the National Pregnancy in Diabetes (NPID) Audit Report 2021 and 2022
https://digitalnhsuk/data-and-information/publications/statistical/national-pregnancy-in-diabetes-audit/2022
